# Effect of two methods and two anaesthetics for local anaesthesia of piglets during castration

**DOI:** 10.1186/s13028-020-00566-8

**Published:** 2021-01-06

**Authors:** Lotte Skade, Charlotte Sonne Kristensen, Mai Britt Friis Nielsen, Line Hummelmose Diness

**Affiliations:** Landbrug & Fødevarer F.m.b.A., SEGES, Axeltorv 3, 1609 Copenhagen V, Denmark

**Keywords:** Behaviour, Lidocaine hydrochloride, Pain, Procaine hydrochloride, Vocalisation

## Abstract

**Background:**

Since January 2019, surgical castration of male piglets must be performed using local anaesthesia, if farmers deliver pigs to the primary exporting slaughterhouses according to the “Danish quality scheme”; a voluntary initiative taken by the Danish pig industry. The approved procedure for local anaesthesia in Denmark is a three-step injection method with procaine. A comparison of lidocaine and procaine with the same concentration and injection methods of local anaesthetics has not previously been studied. The purpose of this study was to investigate the effect of two injection methods and two local anaesthetics on piglets’ avoidance behaviour (vocalisation and resistance movements) as well as the time spent on the procedures. The study included 203 male piglets that were randomly assigned to one of five treatments: 1. Control: Sham-handling without injection of local anaesthesia, 2. Pro3: Procaine injection using a three-step method, 3. Pro2: Procaine injection using a two-step method, 4. Lid3: Lidocaine injection using a three-step method, 5. Lid2: Lidocaine injection using a two-step method. During injection of local anaesthesia and castration, vocalisation was measured using a decibel meter and resistance movements were registrated by video recordings.

**Results:**

During castration, piglets treated with local anaesthesia showed significantly reduced vocalisation and resistance movements and time spent on castration was also significantly reduced compared to the control group. During injection of the local anaesthesia, the piglets had significantly increased vocalisation and resistance movements compared to the control group. Piglets injected with lidocaine had a significantly reduced resistance movement score and a tendency to reduced vocalisation compared to piglets injected with procaine. No differences in avoidance behaviour were found between the injection methods.

**Conclusions:**

The use of local anaesthesia, irrespective of the method and local anaesthetic, was effective in reducing vocalisation and resistance movements during surgery as well as the time spent on castration.

## Background

Globally, most piglets are surgically castrated to eliminate the occurrence of boar taint in the meat and to prevent sexual and aggressive behaviour. It is generally accepted that castration is a painful procedure and compromises piglet welfare. According to EU Council Directive 2008/120/EC of 18 December 2008, castration of piglets is allowed under the condition that it is carried out by a veterinarian or a person trained and experienced in performing the applied techniques with appropriate means and under hygienic conditions. If castration or docking of tails is practised after the seventh day of life, it shall only be performed under anaesthetic and additional prolonged analgesia by a veterinarian. To address the welfare issue of castration representatives from the European pig industry (farmers, meat industry, retailers, scientists, veterinarians and animal welfare NGOs), voluntary agreed in 2010 to focus on a goal to ban surgical castration by the end of 2018. This was stated in the voluntary agreement ‘European Declaration on alternatives to surgical castration of pigs’ [1]. The first step of the declaration stipulated that all piglets should receive prolonged pain relief from January 2012, either alone or in combination with an anaesthetic in the form of a general anaesthetic or a local anaesthetic. Even though the primary goal of the declaration—to abandon surgical castration—was not achieved by the end of 2018, several European countries have made a local agreement to ban surgical castration without analgesia and/or anaesthesia [2].

In Denmark, it has been mandatory for castrated piglets to be treated with prolonged analgesia since 2009, and according to an initiative by the Danish pig industry from January 2019, all male piglets in Denmark should be treated with local anaesthesia prior to castration. In Denmark, the approved method of administering local anaesthesia has been described by the Danish Veterinary and Food Administration [3], and Danish pig producers and their employees are allowed to administer local anaesthetics themselves after having completed a personal mandatory course.

The evaluation of different analgesia and/or anaesthesia protocols is complicated by difficulties in assessing the perception of pain, since pain is a subjective experience and varies between individuals. In the absence of specific parameters measuring pain in pigs, increased avoidance behaviour has been identified as the best indicator of pain [4]. In this study, vocalisation and resistance movements are defined as avoidance behaviour, and both are typical responses that are increased during castration when piglets experience acute pain [4–10]. However, avoidance behaviour requires careful interpretation, because it is not necessarily a specific indicator of pain, since vocalisation and resistance movement also increase when the piglets are handled [4, 7, 10, 11]. The advantage of using vocalisation as an indicator of pain in pigs is that it can be measured objectively by intensity (decibel level) or number of high-frequency calls and has been validated [5].

Several studies have shown an effect of local anaesthesia applied prior to castration [7, 12–17]. However, there is limited research on the most effective injection method of local anaesthesia for male piglets prior to castration [16], and many of the studies do not include a description of the exact injection method (e.g. needle length and direction, place of needle insertion) [18].

Due to the EU regulation, lidocaine can only be used for piglets in accordance with the cascade rule, because a maximum residue limit (MRL) value for lidocaine has not been defined. Since lidocaine provides a better and more rapid onset of local anaesthesia than achieved using procaine [19–21], lidocaine is often used for local anaesthesia of piglets. There are indications that lidocaine has an onset of effect of only three minutes after the injection [22, 23], whereas an effect is achieved after five minutes with procaine [24]. Previous studies that have compared lidocaine and procaine do not seem to have considered the difference in the time of onset for the two drugs [25, 26].

The purpose of this study was to investigate the effect of two injection methods and two local anaesthetics on piglets’ avoidance behaviour (vocalisation and resistance movements) as well as time spent on the procedures in a commercial herd.

## Methods

The study was a field trial approved by the Danish Medical Agency (approval number 2019-05-03-08) and was carried out between October and December 2019.

It was conducted on four separate days over a period of two weeks in a Danish conventional herd with approximately 800 sows producing crossbred (Landrace & Yorkshire × Duroc) piglets.

### Inclusion of piglets

All healthy male piglets, aged three to seven days, were included. Male piglets with anatomical malformations in the groin area were excluded. The male piglets were weighed and randomly assigned to one of five groups (Table 1): (1) Control: Sham-handling without injection of local anaesthesia, (2) Pro3: Procaine hydrochloride injection using a three-step method, (3) Pro2: Procaine injection using a two-step method, (4) Lid3: Lidocaine injection using a three-step method, (5) Lid2: Lidocaine hydrochloride injection using a two-step method.

**Table 1:** Description of the control and four treatment groups combining injection methods and local anaesthetics

Group	Control	Pro3	Pro2	Lid3	Lid2
Local anaesthesia method	SHAM	Three-step	Two-step	Three-step	Two-step
Local anaesthesia drug	–	Procaine	Procaine	Lidocaine	Lidocaine
Volume per testicle (mL)	–	Max 0.5	Max 0.3	Max 0.5	Max 0.3
Time from injection to castration (min)	Min. 3	5	5	3	3

SHAM piglets were placed in the castration bench, the testicles were held in position, but no local anaesthetic was applied (sham-anaesthetised)

### Interventions

Throughout the entire study, all piglets were handled by the same two veterinarians, who were not blinded to the treatment protocol for practical reasons. The administration of the local anaesthetic and the castration were performed by the same veterinarian in every treatment. No other routine treatments (e.g. iron injection or coccidiosis treatment) were given on the day of castration, besides nonsteroidal anti-inflammatory drugs (NSAIDs), which were given to all piglets immediately after castration. Ear tagging of the piglets was carried out the day before castration.

Male piglets were collected by litter in a cart and moved to a quieter room outside the farrowing unit. The piglets were kept in the cart with their litter mates between the local anaesthesia injection and the castration.

The procedure was divided into two phases: phase (1) the sham-handling/local anaesthesia, and phase (2) the castration (Table 1). In phase 1, the piglets in the treatment groups were injected with a local anaesthetic, and the control piglets were handled in the same way, but without injection of the local anaesthetic (sham-handling) [6, 9, 10, 26, 27]. In phase 2, all piglets, regardless of group, were castrated in the same way. During both phases, the piglets were restrained in a castration bench.

### Local anaesthesia injection (Phase 1)

The applied local anaesthetics were either procaine hydrochloride 2% solution (Procamidor Vet., 20 mg/mL, Richter Pharma AG, Austria) in groups Pro3 and Pro2 or lidocaine hydrochloride 2% solution (Xylocain^®^, 20 mg/mL, Aspen Pharma Trading Limited, Ireland) in groups Lid3 and Lid2. Neither of the local anaesthetic solutions contained adrenalin, because vasoconstrictors can modify the effect of the local anaesthetic [21].

The local anaesthetic was applied using an automatic syringe (HSW Eco-Matic, 0.5 mL) with a 25G needle (0.5 mm × 1.6 mm, BD Microlance™ 3, Becton, Dickinson and Company). Needles were changed between each piglet.

The three-step method was applied to the piglets in groups Pro3 and Lid3. When the piglet was restrained on its back in the castration bench, the skin over the testicle was tightened to expose the testicle and hold in position in the scrotum. The needle was inserted directly into the spermatic cord (intrafunicularly), through the skin and testicle. This was achieved by aiming the needle towards a point between the highest point of the shoulder blades on the opposite foreleg. A volume of 0.5 mL of the local anaesthetic was then applied, while the needle was simultaneously being withdrawn in order to distribute the volume of the local anaesthetic evenly in a line from the spermatic cord to the surface of the skin. This was carried out for each testicle.

The two-step method was applied to the piglets in groups Pro2 and Lid2. As with the three-step method, the piglet was restrained in the castration bench, the testicle was held in position and the needle was aimed in direction of the highest point of the shoulder blades on the opposite foreleg. The needle was inserted to only half its length, so that the tip of the needle was positioned intratesticularly. A volume of 0.3 mL of the local anaesthetic was then carefully applied, while the needle was simultaneously being withdrawn, also in order to distribute the volume of the local anaesthetic evenly in a line from the testicles to the surface of the skin. This was carried out for each testicle.

### Castration (Phase 2)

Phase 2 was the castration, which was performed in accordance with established procedures under housing unit conditions, as described in Prunier [28], using a scalpel to make the skin incision and cut the spermatic cord.

Due to differences in the time of onset for each drug, the time between the phases varied. The piglets stayed in the cart between injection of local anaesthesia and the castration. Control piglets were castrated at least three minutes after phase 1. The piglets treated with lidocaine (Lid3 and Lid2) were castrated after three minutes, while the piglets treated with procaine (Pro3 and Pro2) were castrated after five minutes.

### Vocalisation

During both phases, vocalisation was measured using a decibel meter (2237 Controller, Integrating Sound Level Meter, from Brüel and Kjær), which was placed 10 cm from the piglet’s snout. All measurements were conducted by the same veterinarian and both the average decibel level (dB(A)_avg_) and the maximum decibel level (dB(A)_max_) were measured. The decibel levels were measured for a period of ten seconds for phase 1 and 30 s for phase 2, because these were the estimated time required to perform the procedures.

### Resistance movements

A camera was used to record the handling of the piglets in phase 1 and phase 2 in order to facilitate the evaluation of the resistance movements and to allow them to be evaluated by an observer. The observer was blinded to the treatment groups of the piglets (whether the piglets were anaesthetized or not and which method was applied) during the evaluation of all the video recordings from phase 2, which were the first video recordings to be evaluated. During evaluation of the video recordings from phase 1, the observer could not be blinded because the differences in the injection methods were visible on the video recordings.

The resistance movements were evaluated as follows: level 1 = no intensity (no movement), level 2 = low intensity (one to three movements of the foreleg), and level 3 = high intensity (> three movements of the foreleg, hind leg and/or body). The ranks are modified after the study by Leidig et al. [7]. Every piglet was evaluated four times in each phase (Table 2), and the average of these four evaluations resulted in the resistance movement score. The resistance movement score could thus assume a decimal number between 1 and 3. For example, during phase 1, the resistance movements in each of the four time intervals were evaluated to be 1, while the resistance movements during phase 2 were evaluated to be 1, 3, 3, and 2 in the four time intervals, respectively. The resistance movement scores for phases 1 and 2 were therefore 1.0 and 2.25, respectively.

**Table 2:** Definition of time intervals during phase 1 and phase 2, where resistance movements were scored

Time interval	Phase 1 (local anaesthesia)	Phase 2 (castration)
1	Injection in testicle 1	Incision in scrotum above testicle
2	Between injections	Extraction of testicles
3	Injection in testicle 2	Removal of testicles by cutting the spermatic cord
4	Five seconds after the last injection	Five seconds after the end of castration

### Time consumption

The time spent on the injection of local anaesthesia in phase 1 and castration in phase 2 was evaluated using the video recordings. In phase 1, the time was measured from the insertion of the needle in the first testicle and until the needle had been removed from the piglet after the injection in the other testicle. In phase 2, the time was measured from the incision in the skin until both testicles had been removed.

### Statistical analysis

The study was designed as a two-factor trial (drug and injection method) to show a difference in vocalisation at 10 dB(A) between the two factors. In a preliminary trial, the spread was measured as 20 dB(A). In a two-samples t-test with alpha set at 0.025, 80 animals are required per factor, which is 40 per group, resulting in a total of 200 (5 × 40).

The sound level (decibel) was calculated as both the average and maximum during the measuring period. Both parts were analysed in a linear model, with drug and method as explanatory variables, and the piglets’ weight as covariate and litter as random effect.

The resistance movements were analysed as binary outcomes, with level 1 (no resistance movement) compared to levels 2 and 3 (resistance movements) in a logistic model with a link = logit function, with model and drug as response variables, and the piglet’s weight as covariate and repeated measurements in the group. The resistance movement score and time consumption were analysed in a linear model, with drug and method as explanatory variables, the piglet’s weight as covariate and repeated measurements in the group.

Separate analyses were conducted for phase 1 and phase 2. These were performed using SAS^®^ software, Version 9.4 (Copyright © 2014, SAS Institute Inc, Cary, NC, USA) and applying a significance level of 5%.

## Results

A total of 215 piglets from 35 different litters were initially ear tagged. Five piglets were excluded due to ill-thrift or anatomical malformations in the groin area. Seven piglets were excluded due to incomplete data acquisition.

Vocalisation was measured during phases 1 and 2 (Table 4). Video recordings for subsequent evaluation of resistance movements during phase 1 and phase 2 were recorded for 150 piglets (Table 5). Initially, recordings were made for all piglets, but recordings from 53 piglets were lost because of technical issues with file transfer to computer.

Table 3 shows the distribution of the piglets among the groups and descriptive statistics regarding their weights.

**Table 3:** Weight of piglets in groups with respect to measurement of vocalisation and number of resistance movements

Group	Control	Pro3	Pro2	Lid3	Lid2
Vocalisation					
N	43	42	38	40	40
Weight, kg	2.38	2.36	2.31	2.33	2.48
Standard deviation	0.52	0.48	0.57	0.47	0.52
Resistance movements					
N	31	30	27	29	33
Weight, kg	2.40	2.36	2.32	2.37	2.53
SD	0.52	0.51	0.56	0.50	0.53

*Pro3* treatment with three-step injection with procaine, *Pro2* treatment with two-step injection with procaine, *Lid3* treatment with three-step injection with lidocaine, *Lid2* treatment with two-step injection with lidocaine

### Vocalisation

In phase 1, the dB(A)_avg_ and dB(A)_max_ were higher in the treatment groups than the control group (P = 0.02 and P < 0.01, respectively) (Table 4). In phase 2, the dB(A)_avg_ and dB(A)_max_ were lower in the treatment groups than the control group (P < 0.01).

**Table 4:** Vocalisation during phase 1 and phase 2

Group	Groups					SE	P values		
	Control	Pro3	Pro2	Lid3	Lid2		Treatment/control	Two-step/three-step	Lidocaine/procaine
Phase 1 (local anaesthesia/handling)									
dB(A)_avg_	67	74	75	71	70	2.1	0.02	0.98	0.07
dB(A)_max_	86	94	96	92	91	2.1	< 0.01	0.99	0.07
Phase 2 (Castration)									
dB(A)_avg_	77	67	70	67	68	1.5	< 0.01	0.08	0.44
dB(A)_max_	101	92	94	92	94	1.5	< 0.01	0.19	0.77

*SE* standard error, *dB(A)*_*avg*_ mean decibel(A), dB(A)_max_ decibel(A), *Pro3* treatment with three-step injection with procaine, *Pro2* treatment with two-step injection with procaine, *Lid3* treatment with three-step injection with lidocaine, *Lid2* treatment with two-step injection with lidocaine

The lowest dB(A)_max_ was measured in the control piglets during phase 1. In contrary, the highest dB(A)avg and dB(A)max was measured in the control piglets during phase 2. During phase 2, the piglets in groups Pro3 and Lid3 vocalised at the same dB(A)_avg_ level as the control piglets when they were sham-handled during phase 1.

Local anaesthesia administered by the two-step method (Pro2 and Lid2) or three-step method (Pro3 and Lid3) caused no difference in piglets’ vocalisation, either during phase 1 or phase 2.

Injection with procaine (Pro3 and Pro2) tended to cause a higher vocalisation compared to injection with lidocaine (Lid3 and Lid2) in phase 1, but there was no difference in piglets’ vocalisation during phase 2 (Table 4).

### Resistance movements

In phase 1, a significantly lower resistance movement score was found for the control group compared with the treatment groups (Table 5). Moreover, the piglets had a significantly higher resistance movement score during injection with procaine compared to injection with lidocaine based on the resistance movement score (Pro2 and Pro3 compared with Lid2 and Lid3) in phase 1 (P = 0.02).

**Table 5:** Number of piglets without resistance movements, resistance movement score and time consumption during phase 1 and phase 2

	Groups					SE	P values		
	Control	Pro3	Pro2	Lid3	Lid2		Treatment/control	Two-step/three-step	Lidocaine/ procaine
Phase 1 (local anaesthesia/handling)									
Testicle 1, % piglets without resistance movements (level 1)	98	67	59	65	76	1.0	< 0.01	0.82	0.31
Testicle 2, % piglets without resistance movements (level 1)	90	57	56	69	70	1.0	< 0.01	0.99	0.13
Resistance movement score	1.2	1.7	1.9	1.6	1.5	0.1	< 0.01	0.88	0.02
Time consumption, sec	3.2	6.1	5.7	5.5	5.1	0.3	< 0.01	0.07	0.03
Phase 2 (castration)									
Incision in skin, % piglets without resistance movements	20	67	65	79	63	8	< 0.01	0.27	0.59
Removal of testicles, % piglets without resistance movements	6	29	31	36	52	9	< 0.01	0.30	0.16
Resistance movement score	2.3	1.8	1.9	1.8	1.7	0.1	< 0.01	0.99	0.15
Time consumption, sec	15	14	14	13	14	1.4	0.04	0.34	0.36

*SE* standard error, *Pro3* treatment with three-step injection with procaine, *Pro2* treatment with two-step injection with procaine, *Lid3* treatment with three-step injection with lidocaine, *Lid2* treatment with two-step injection with lidocaine

During phase 2, a significantly increased number of piglets with resistance movements during incision in the skin and removal of testicles (P < 0.01) and a significantly higher resistance movement score (P < 0.01) were found for the control group compared to the treatment groups. No differences in the number of piglets with resistance movements during incision in the skin and removal of testicles or the resistance movement score were found when comparing the four different treatment groups (Pro3 and Lid3 compared with Pro2 and Lid2; Pro3 and Pro2 compared with Lid3 and Lid2; Table 5).

### Time consumption

It took an average of 5.6 s to administer the local anaesthetic per piglet in the treatment groups independently of the method or drug used for the injection (Table 5). Injecting the lidocaine was faster than injecting the procaine (P = 0.03), and the two-step method tended to be faster than the three-step method (P = 0.07; Table 5). On average, the castration of the piglets in the treatment groups took 14 s to perform, which is one second faster than the castration of the control piglets (P < 0.01).

## Discussion

The results showed that local anaesthesia applied with a 25G needle reduced avoidance behaviour (vocalisation and resistance movement) during castration of piglets in the treatment groups compared to control piglets. Local anaesthesia should therefore continue to be used as a routine procedure every time male piglets are castrated.

Several studies have found that male piglets locally anaesthetised by injection experience considerably less pain during castration, assessed by decreased vocalisation and resistance movements compared to piglets castrated without local anaesthesia [6, 7, 12, 14, 15, 29]. Only few studies have assessed the pain during injection of the local anaesthesia by behaviour or physiologic measures [7, 12, 13, 25]. To our knowledge, this study is the first to investigate injected local anaesthetics using the same injection methods and drug concentrations.

The two-step injection method is possibly more convenient to use than the three-step injection method, as it tended to be faster and used a reduced volume of local anaesthetic, though it had the same analgesic effect as the three-step injection method. A comparable study of two injection methods [13] did not find a difference in physiological indicators for stress or pain (blood pressure, pulse rate or EEG) when the piglets were injected intrafunicularly and intratesticularly, and therefore they recommended intratesticular injection.

Irrespective of which local anaesthetic used in the study, no difference was found on the locally anaesthetised piglets’ avoidance behaviour during castration. This is probably because the difference in the local anaesthetic’s time of onset were considered. In the herdsmen’s daily work, other tasks such as ear tagging, treatment with coccidiostats, tail docking and vaccination are usually carried out in the waiting period between the administration of local anaesthesia and the castration. Herds requiring only few tasks to be carried out in this waiting period would benefit from using a local anaesthetic with a shorter time of onset.

We used a much finer needle (25G) than usually for local anaesthesia injection of piglets before castration, because smaller needle diameters have shown to minimise the pain caused by the needle insertion in piglets [18] and in humans [30]. Besides the pain caused by insertion of the needle, pain of injection may also result from the tension or pressure created in the injected tissue, which depends on several factors, such as the injection volume, speed, tissue density, and the viscosity of products [31, 32]. Efforts to minimise excessive tension in the tissue were made by limiting the volume of the local anaesthetic applied and dispersing the volume evenly across a larger area by injecting at the same time as withdrawing the needle. To standardise the injection speed as much as possible, all injections were carried out by the same veterinarian. As we were not able to fully prevent discomfort of injection, it should be investigated if the discomfort of injection can be further reduced by adjusting injection volumes, speeds, and needle sizes.

It is remarkable that the level of vocalisation during the castration of piglets treated with local anaesthetics is equal to the vocalisation of the control piglets when being sham-handled. This finding is in accordance with results of previous studies [6, 15, 29, 33]. Based on this, it can be considered that the level of the piglet’s discomfort is identical, irrespective of whether it is restrained or castrated under local anaesthesia.

The reduction in vocalisation, which was 10 dB(A)_avg_ during castration (phase 2) when comparing treated piglets with control piglets, corresponds to a 90% reduction of the sound level, since sound is measured on a logarithmic scale. However, the difference between the measurement of sound and the perception of sound means that the reduction in vocalisation cannot necessarily be heard during routine activities in a commercial herd [34]. Reducing the level by 3 dB corresponds to reducing the sound pressure by half, but it is necessary to reduce the level by at least 10 dB for the perception of the sound to be reduced by half [35].

Increased vocalisation was observed during the administration of the local anaesthetic, although it was both lower and of less duration than the vocalisation during castration. The increased vocal responses during intrafunicular or intratesticular injection of anaesthetics correspond to results observed in other studies [6, 7, 12, 13, 25], where behaviour or nociceptive responses were found to be increased during injection, but also indicate that the level of discomfort experienced during local anaesthesia is lower than the level of pain experienced by control piglets during castration.

Regarding resistance movements, the locally anaesthetised piglets had a lower resistance movement score than the control piglets during castration, substantiating that locally anaesthetised piglets experience less pain during castration.

The scrotum, testicles and adjacent structures have a complex nerve supply [28]. The nerve supply to the scrotum originates from branches of the pudendal nerve and cutaneous nerves. The testicles and epididymis are innervated by nerves from the testicular plexus which travel along with the gonadal vessels in the spermatic cord. The innervation of the cremaster muscle and the vaginal tunic originates from the genitofemoral nerve, from which the genital branch of the nerve passes through the inguinal ring with the spermatic cord to the specified structures [36, 37]. It is known that pulling and cutting of the spermatic cord and cremaster muscle causes most pain to piglets when castrated [9, 12, 27], requiring a sufficient local anaesthesia of both structures. Unfortunately, Ranheim et al. [22] showed, when local anaesthesia was injected intratesticularly, it dispersed proximal in the spermatic cord where the nerve supply to the testicle and epididymis is present, but not readily dispersed through the vaginal tunic to the cremaster muscle. Therefore, an inadequate local anaesthesia of the cremaster muscle and the vaginal tunic is probably the reason that resistance movements were observed from 48–71% of the anaesthetised piglets while the testicles were removed in this study. But even though most of the piglets made resistance movements, the resistance movement score and vocalisation had a lower intensity indicating less pain, when the testicles were removed compared to the control piglets. Further investigations should be conducted to determine how to prevent pain when severing the cremaster muscle.

In phase 1, piglets injected with procaine were evaluated as having a higher resistance movement score, indicating that injecting procaine caused greater discomfort in the piglets than injecting lidocaine. While Zankl et al. [26] did not find a difference between injection of anaesthetics and methods evaluated by serum cortisol concentrations, Hoffmann et al. [25] and Rauh et al. [12] found a difference corresponding to our results, indicating that procaine causes greater discomfort in the piglets.

The resistance movements were evaluated by a single observer to avoid inter-observer bias. The intensity was only evaluated in three ranks, based on numbers of movements of the legs, which is very easy to observe. Before starting to evaluate the video recordings from phase 2, the observer was trained to recognise the three ranks, and before the observer moved on to evaluation of video recordings from phase 1, the training was repeated to improve intra-observer reliability.

The time spent in the castration bench could have an impact on the occurrence of resistance movements. Apart from the Lid3 group, an increased number of piglets were observed with resistance movements from the injection in the first testicle to the injection in the second testicle during phase 1. Since this was also observed in the control group, where the piglets were only subjected to sham-handling, it indicates that the piglets’ reaction increases with the time spent lying in the castration bench.

Injecting lidocaine was 0.6 s faster than injecting with procaine, regardless of the method used and the volume injected. Since the piglets also moved less when being injected with lidocaine, it is hypothesised that injection with lidocaine causes less pain than injection with procaine (Table 5). However, it is also a possibility that the decreased injection time caused a decreased resistance movement, because the piglet had less time to move.

It is recommended to investigate how different concentrations and/or the addition of adrenaline to the local anaesthetic can affect the time of onset and the duration time when castrating male piglets to ensure that the processing time is used effectively to provide maximum benefit of the local anaesthesia in the piglets.

This study focused exclusively on the effect of local anaesthetics during injection and castration. Other studies have shown that piglets’ behaviour and physiological parameters are adversely affected for up to five days after castration without local anaesthesia [26, 38, 39] and that local anaesthesia can reduce the adverse effect immediately after castration [6, 14]. However, the best effect is achieved through a combination of local anaesthesia and NSAIDs [40]. Procaine and lidocaine are short-time-acting local anaesthetics, and, according to the SPCs for the products available in Denmark, the duration time of procaine for veterinary use is 30–45 min [41], while the duration time of lidocaine for local infiltration in humans is at least 90–180 min [42]. It should be further investigated if or how the longer duration of lidocaine action may cause better long term pain relief.

Furthermore, an increased mortality of 1.6–2.7% has been found between castrated piglets and entire males [43, 44]. This is assumed to be due to the increased risk of infection, castration injuries and/or other complications during healing (e.g. excessive scar tissue formation), although it has only been investigated to a limited extent [6]. It would thus be interesting to investigate if the local anaesthetic drugs affect the risk of infection rate and mortality and how the local anaesthetics are distributed in the tissue, how the distribution is affected by different methods and how different work procedures affect the piglets’ behaviour.

## Conclusions

Local anaesthesia results in significantly fewer pain responses during castration measured in terms of vocalisation and resistance movements in locally anaesthetised piglets compared with control piglets.

Injection with lidocaine caused less discomfort in the piglets than injection with procaine. In relation to castration, no differences were found between the use of procaine and lidocaine.

Similarly, no significant difference in measurements of vocalisation, resistance movement or time consumption was found between the use of the two-step or three-step method for local anaesthesia injection. Thus, it might be beneficial to use the two-step method, since it tends to be faster and uses a smaller amount of local anaesthetic.

It takes less time to castrate locally anaesthetised piglets, irrespective of the local anaesthetic or method used.

## Data Availability

The datasets used and analysed during the current study are available from the corresponding author on reasonable request.
